# Apolipoprotein E Genotype, Meat, Fish, and Egg Intake in Relation to Mortality Among Older Adults: A Longitudinal Analysis in China

**DOI:** 10.3389/fmed.2021.697389

**Published:** 2021-07-20

**Authors:** Xurui Jin, Shangzhi Xiong, Changzheng Yuan, Enying Gong, Xian Zhang, Yao Yao, Yu Leng, Zhangming Niu, Yi Zeng, Lijing L. Yan

**Affiliations:** ^1^Global Health Research Center, Duke Kunshan University, Kunshan, China; ^2^Mindrank AI Ltd, Hangzhou, China; ^3^The George Institute for Global Health, University of New South Wales, Sydney, NSW, Australia; ^4^Department of Big Data and Health Science, School of Public Health, School of Medicine, Zhejiang University, Hangzhou, China; ^5^School of Population and Global Health, University of Melbourne, Melbourne, VIC, Australia; ^6^Center for Healthy Aging and Development Studies, National School of Development, Peking University, Beijing, China; ^7^Center for the Study of Aging and Human Development and Geriatrics Division, Medical School of Duke University, Durham, NC, United States; ^8^Duke Global Health Institute, Duke University, Durham, NC, United States; ^9^The George Institute for Global Health, Beijing, China; ^10^Department of Preventive Medicine, Feinberg School of Medicine, Northwestern University, Chicago, IL, United States

**Keywords:** APOE genotype, healthy aging, Chinese older adults, diet, longitudinal study

## Abstract

**Introduction:** The interactions between apolipoprotein E (*APOE*) genotype and diet pattern changes were found significant in several trials, implying that *APOE* gene may modify the effect of animal protein-rich food on health outcomes. We aim to study the interaction of *APOE* genotype with the effect of meat, fish and egg intake on mortality.

**Methods:** This population-based study enrolled 8,506 older adults (mean age: 81.7 years, 52.3% female) from the Chinese Longitudinal Healthy Longevity Study. The intake frequency of meat, fish and egg was assessed by 3-point questions at baseline. Cox regression was conducted to calculate the hazard ratios for all-cause mortality of intake levels of meat, fish and egg. The analyses were stratified by *APOE* genotype and sex. The analyses were performed in 2020.

**Results:** In the multivariable-adjusted models, meat and fish intake was associated with all-cause mortality (high vs. low intake: meat: HR: 1.14, 95% CI: 1.01, 1.28; fish: HR: 0.83, 95% CI: 0.73, 0.95). *APOE* genotype have significant interactions with meat and fish intake (*P*s < 0.05). Compared with low fish intake, high fish intake was associated with lower risk of mortality (HR: 0.74, 95% CI: 0.56–0.98) only among the *APOE* ε4 carriers. High meat intake was significantly associated with higher risks of mortality (HR: 1.13, 95% CI: 1.04–1.25) only among the *APOE* ε4 non-carriers. The interactive relationship was restricted among the male. No significant findings were observed between egg and mortality among carriers or non-carriers.

**Conclusions:** Among Chinese older adults, the significance of associations of mortality with reported meat or fish intake depended on APOE-E4 carriage status. If validated by other studies, our findings provide evidence for gene-based “precision” lifestyle recommendations.

## Introduction

During the last 50 years, the relationship between dietary factors and mortality has been investigated in many population studies. It has been demonstrated that diet is one of the major determinants of longevity (1). However, the effects of meat, fish, and egg consumption on mortality are not consistent across studies and considerable interindividual heterogeneity has been noted (2–4). For instance, a study with 134,290 Chinese adults suggested that a high intake of meat was associated with higher mortality (2), while a pooled analysis including 29,721 Asians failed to provide similar evidence (5). One potential explanation for this inconsistency may be the modifying effect of genetic factors across different individuals. Currently, accumulating evidence suggested that genetic factors, as one of the main sources of human variation, interacted with multiple dietary factors in determining health outcomes (6–8).

Among numerous genetic factors, the Apolipoprotein E *(APOE)* gene has a high probability to interact with the intake of meat, fish, and egg on mortality. In the past few years, genome-wide investigations have identified several variants associated with mortality and longevity. Interestingly, nearly all such studies in different population have identified *APOE* gene as one of the genetic factors associated with mortality or longevity (9). Moreover, several existing trials found that *APOE* gene played an important role in lipid metabolism and is associated with blood lipid level (10–12). Specifically, *APOE* ε4 carriers had a faster response in several lipid-related biomarkers to the same dietary interventions compared with the ε4 non-carrier. Such existing evidence increased the biological plausibility for the possible interactions between *APOE* genotype and meat, fish and egg intake on human's health. However, their possible interactive effects on people's long-term health outcomes, such as mortality, remains unclear.

In this study, we hypothesized that the relation between meat, fish and egg consumption and mortality may be modified by *APOE* ε4 genotype among Chinese older adults. By testing this hypothesis, we expect to contribute meaningful implications to the development of personalized diet recommendation for healthy aging.

## Methods

### Study Sample

The present study uses data from the Chinese Longitudinal Healthy Longevity Study (CLHLS), which is a longitudinal study on Chinese older adults since 1998 with follow-up surveys every 2–3 years. The CLHLS surveys were conducted in randomly selected cities and counties that accounted for half of all cities and counties in 23 out of 31 provinces of China, which covered over 85% of China's population. Based on sex and place of residence (i.e., living in the same street, village, city, or county) for a given centenarian, randomly selected octogenarians and nonagenarians were also sampled. More details of the study have been published elsewhere (13). Ethic approval was obtained from the Research Ethics Committees of Peking University and Duke University. All participants or their legal representatives signed written consent forms in the baseline and follow-up surveys.

Our analyses included 8,552 elderly aged 65 years or over from five waves of CLHLS (2000, 2002, 2005, 2008, and 2012) who had both genotypic and phenotypic measurements. At baseline, a questionnaire was used to assess the frequency of meat, fish and egg intake and other co-variates. Follow-up surveys were performed every 2 or 3 years until 2014. We excluded 46 participants due to missing values in dietary measurement, resulting in a final sample of 8,506. There were no familial/kinship relations among the participants within and across different waves. The analysis was performed in 2020.

### Genotyping

A customized chip was designed and used to genotype 287,898 SNPs related with longevity, chronic disease, or health markers (14). The genotyping was done by the Beijing Genomics Institute, and the CLHLS genetics study's genotyping quality control procedures have been reported elsewhere (15). We used rs429358 and rs7412 genotype to determine the *APOE* genotypes and the *APOE* genotypes were further grouped by whether or not carrying the ε4 allele.

### Data on Mortality

Vital status and date of death were collected from officially issued death certificates when available or otherwise from the next-of-kin or local residential committees who were familiar with the decedents. Duration of follow-up was calculated by the time interval between the first interview date and date at death. Survivors at the last wave (2012) were censored at the time of the last survey.

### Dietary Assessment

Self-reported information on dietary intake habits was collected by trained interviewers at each wave. The respondents were asked to report the intake frequency for ten different food categories at present (baseline) and at 60 years old, including fresh fruits, vegetables, meat, fish, bean products, tea, garlic, egg, sugar, and salt-preserved vegetables. Each food's frequency of intake was reported as three categories: “almost every day,” “sometimes or occasionally,” or “rarely or never.” The present study focused on the frequency of intake of meat, fish, and egg at baseline. In previous studies based on the same database, the dietary intake variables have been found to significantly relate with cognitive function (16), frailty (17), and mortality (18), which supported the validity of the dietary assessment method.

### Statistical Analysis

Baseline characteristics were presented as mean (continuous variables) or frequency distribution (categorical variables). ANOVA for continuous variables and chi-square tests for categorical variables were applied to compare the differences between *APOE* ε4 carriers and non-carriers. Three Cox proportional hazard models were performed to examine the relationship of mortality with meat, fish, and egg intake separately. Model 1 adjusted for demographic variables: age, sex, education, marital status, ethnicity, living arrangement, residence, and occupation and other dietary factors; Model 2 included all variables in Model 1 and added lifestyle variables (i.e., tobacco smoking status, alcohol drinking status, and current physical activity) and abnormal body weight (i.e., higher than 95% of total population or lower than 5%); Model 3 further adjusted for activities of daily living, leisure activity score (continuous), cognitive function measured by the Mini Mental State Examination (MMSE) score (continuous), and eight types of self-reported diseases (i.e., chronic obstructive pulmonary disease, tuberculosis, cancer, cataract, glaucoma, diabetes, stroke and, cardiovascular disease). We examined the association between mortality with meat, fish and egg intake with these three models in all participants and in each sex subgroup.

We further examined whether the association between meat, fish and egg intake and mortality differed by *APOE* ε4 genotype in all participants and in each sex subgroup. In the subgroup analysis, for each food type, “almost every day” was defined as “high intake,” while “Sometimes or occasionally” and “rarely or never” was merged into “low intake.” The significance of multiplicative interactions between *APOE* ε4 genotype and meat, fish and egg intake for all-cause mortality was tested by adding cross-product terms in Cox regression models.

We conducted five sensitivity analyses to check the robustness of the main results: (1): excluded deaths in the first year after the follow-up survey, as these early decedents may suffer from serious conditions that might confound the relationship; (2) excluded participants with baseline comorbidity (including heart disease, stroke, respiratory disease, and cancer); (3) performed the same analysis on data from the 2008 and 2012 waves, but replaced the body weight variable with body mass index (BMI, height was firstly measured at the 2008 wave); (4) excluded participants who had different dietary patterns of meat, fish and egg intake between the baseline wave and when they were 60 years old (e.g., high intake at 60 years old and low intake at baseline, or the other way around); and (5) excluded participants with MMSE score lower than 16 (cut-off point for severe cognitive impairment) to reduce the recall bias (19).

STATA version 14.0 (Stata Corp, College Station, TX, USA) for Windows was used for data management and analysis.

## Results

### Baseline Characteristics of Participants

Table 1 shows the baseline characteristics of our participants (*n* = 8,506). Our study population had a mean age of 81.7 (standard deviation = 11.7) years with 52.3% being female, and there were 1,640 *APOE* ε4 allele carriers (19.3%) and 6,866 *APOE* ε4 non-carriers (80.7%). Of note, the APOE ε4 allele carriers in our study had significantly younger age than non-carriers (mean age 80.8 vs. 81.9 years, *P* < 0.001). Further descriptions of our study population stratified by sex are included in Supplementary Table 1.

**Table 1:** Characteristics of the 8,506 participants by *APOE* genotype.

**Characteristics^a^**	**Total sample *N =* 8,506**	***APOE*** **Genotype^b^**		
		**Carrier *N =* 1,640**	**Non-carrier *N =* 6,866**	***P*-value^c^**
**Age**, mean ± SD	81.7 ± 11.7	80.8 ± 11.2	81.9 ± 11.7	<0.001
**Sex**				0.068
Men	4,055 (47.7)	815 (49.7)	3,240 (47.2)	
Women	4,451 (52.3)	825 (50.3)	3,626 (52.8)	
**Residence**				0.14
Urban	2,906 (34.2)	586 (35.7)	2,320 (33.8)	
Rural	5,600 (65.8)	1,054 (64.3)	4,546 (66.2)	
**Main occupation before age 60**				0.45
Non-manual	652 (7.7)	133 (0.81)	519 (7.6)	
Manual	7,854 (92.3)	1,507 (91.9)	6,347 (92.4)	
**Education background (school years)**				0.48
None (0)	4,910 (57.7)	934 (57.0)	3,976 (57.9)	
Ever (≥1)	3,596 (42.3)	706 (43.0)	2,890 (42.1)	
**Marital status**				0.10
Married (spouse alive)	3,685 (43.3)	740 (45.1)	2,945 (42.9)	
Others^d^	4,817 (56.6)	899 (54.8)	3,918 (57.1)	
**Ethnicity**				0.62
Han	7,975 (93.8)	1,542 (94.0)	6,433 (93.7)	
Others (minority)	531 (6.2)	98 (6.0)	433 (6.3)	
**Living arrangement**				0.14
With household member	7,118 (83.7)	1,392 (84.9)	5,726 (83.4)	
Alone	1,388 (16.3)	248 (15.1)	1,140 (16.6)	
**Impaired activity of daily living^e^**				0.57
Yes	1,109 (13.0)	207 (12.6)	902 (13.1)	
No	7,395 (86.9)	1,433 (87.4)	5,962 (86.8)	
**Leisure activities^f^**, mean ± SD	12.2 ± 2.9	12.4 ± 2.9	12.1 ± 2.9	0.008
**MMSE score**, mean ± SD	24.0 ± 8.1	24.2 ± 7.9	24.0 ± 8.2	0.25
**Tobacco smoking status**				0.043
Ever	2,879 (33.8)	590 (36.0)	2,289 (33.3)	
Never	5,627 (66.2)	1,050 (64.0)	4,577 (66.7)	
**Alcohol drinking status**				0.45
Ever	2,518 (29.6)	498 (30.4)	2,020 (29.4)	
Never	5,988 (70.4)	1,142 (69.6)	4,846 (70.6)	
**Abnormal body weight**				
No	7,844 (92.2)	1,530 (93.3)	6,314 (92.0)	0.070
Yes	662 (7.8)	110 (6.7)	552 (8.0)	
**Regular physical activity**				0.92
No	5,901 (69.4)	1,136 (69.3)	4,765 (69.4)	
Yes	2,605 (30.6)	504 (30.7)	2,101 (30.6)	
**Meat intake**				0.38
Rarely or never	1,084 (12.7)	221 (13.4)	863 (12.6)	
Occasionally	4,616 (54.3)	893 (54.5)	3,723 (54.2)	
Everyday	2,806 (33.0)	526 (32.1)	2,280 (33.2)	
**Fish intake**				0.56
Rarely or never	1,924 (22.6)	380 (23.2)	1,544 (22.5)	
Occasionally	5,299 (62.3)	1,005 (61.3)	4,294 (62.5)	
Everyday	1,283 (15.1)	255 (15.5)	1,028 (15.0)	
**Egg intake**				0.66
Rarely or never	1,021 (12.0)	204 (11.9)	817 (12.4)	
Occasionally	4,120 (48.4)	808 (48.2)	3,312 (49.3)	
Everyday	3,365 (39.6)	628 (39.9)	2,737 (38.3)	

*MMSE, Mini-Mental State Examination*.

a *Numbers shown are N (%) unless otherwise noted*.

b *The APOE genotype was determined by rs429358 and rs7412 genotype and the APOE genotypes were further grouped by whether or not carrying the ε4 allele*.

c *ANOVA for continuous variables and chi-square tests for categorical variables were applied to compare the differences between APOE ε4 carriers and non-carriers*.

d *Other marital status includes separated, widowed, divorced, never married*.

e *Activity of daily living: assessed by six self-reported questions: “Do you need assistance in bathing/dressing/toileting/transferring/eating/continence?.” Impaired ADL was defined as if the participants answered “Yes” for any of those questions*.

f *Leisure activity score: eight activities are assessed: visiting neighbors, shopping, cooking, washing clothes, walking 1 km, lifting 5 kg, crouching and standing up three times, and taking public transportation. We scored each activity 1 for “never,” 2 for “sometimes” 3 for “almost every day.” The score ranged from 5 to 21 with a higher score indicating more leisure activities*.

### Association of Meat, Fish, and Egg Intake With Mortality

The median duration of follow-up was 5.46 years. During a total of 48,800 person-years' follow-up, 3,210 deaths were documented, accounting for 37.7% of the total population. Table 2 shows the associations between meat, fish, and egg intake and all-cause mortality by one unadjusted model and three adjusted models. In Model 1 (adjusted for demographic variables only) and Model 2 (adjusted for variables in Model 1 and lifestyle variables), the associations between meat intake and risk of all-cause mortality were not significant. The association between meat consumption and mortality reached significance in Model 3 after further adjusting for disability, cognitive function, leisure activities and chronic conditions, where higher (everyday) meat intake was associated with higher risk of mortality compared with low (rarely or never) meat intake (HR: 1.14, 95% CI: 1.01, 1.28). In Model 3, compared with low (rarely or never) fish intake, high (daily) fish intake was associated with 17% (95% CI: 5%, 27%) lower risk of mortality. No significant associations were observed between egg intake and all-cause mortality.

**Table 2:** Hazard ratio and 95% Confidence Interval (CI) of meat, fish, and egg on mortality.

**Groups**	**Participants (*N*)**	**Incidence of mortality, per 1,000 person-years (95% CI)**		**Hazard Ratio (95% CI)**		
			**Unadjusted**	Model 1*	Model 2**	Model 3***
**Meat intake**						
Rarely or never	1,084	65.6 (59.9, 72.0)	Ref.			
Occasionally	4,616	69.1 (57.3, 64.7)	1.07 (0.96, 1.20)	1.04 (0.81, 1.16)	1.02 (0.92, 1.15)	1.04 (0.93, 1.16)
Everyday	2,806	60.9 (65.6, 71.4)	1.03 (0.92, 1.17)	1.08 (0.95, 1.22)	1.08 (0.95, 1.22)	1.14 (1.01, 1.28)
**Fish intake**						
Rarely or never	1,924	71.9 (67.3, 76.9)	Ref.			
Occasionally	5,299	68.5 (65.5, 71.6)	0.92 (0.84, 1.01)	0.90 (0.82, 0.99)	0.91 (0.83, 0.99)	0.95 (0.87, 1.04)
Everyday	1,283	48.5 (44.1, 53.4)	0.65 (0.57, 0.74)	0.78 (0.69, 0.89)	0.78 (0.69, 0.90)	0.83 (0.73, 0.95)
**Egg intake**						
Rarely or never	1,021	60.6 (55.0, 66.8)	Ref.			
Occasionally	4,120	68.7 (65.4, 72.3)	1.17 (1.05, 1.32)	1.07 (0.98, 1.16)	1.09 (0.97, 1.15)	1.07 (0.99, 1.18)
Everyday	3,365	64.2 (60.8, 67.9)	1.15 (1.03, 1.30)	0.98 (0.87, 1.10)	1.03 (0.86, 1.09)	1.02 (0.90, 1.24)

Model 1* adjusted for demographic variables: age, sex, education, marital status, ethnicity, living arrangement, residence, and occupation and other nine kind of dietary components; Model 2** added lifestyle variables (tobacco smoking status, alcohol drinking status and current physical activity) and abnormal body weight for adjustment; Model 3*** further adjusted for activity of daily living, leisure activity score, The Mini-Mental State Examination (MMSE) score, and eight kinds of self-reported diseases (COPD, tuberculosis, cancer, cataract, glaucoma, diabetes, stroke and cardiovascular disease) based on Model 2.

### Interaction of APOE ε4 Genotype With Meat, Fish, and Egg Intake

**Figure 1:**
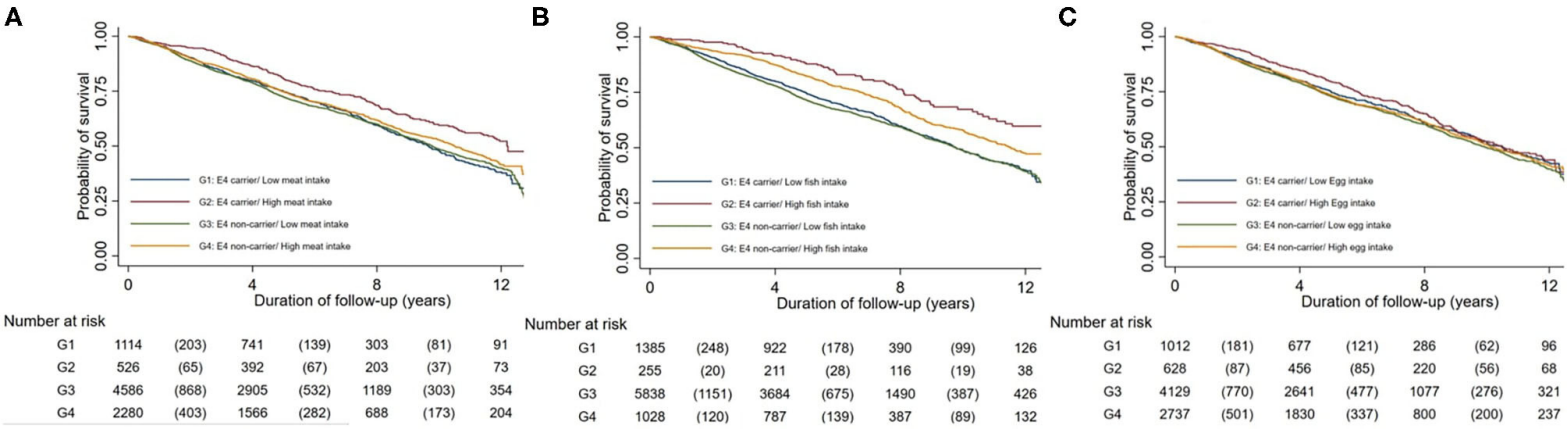
Kaplan-Meier estimates of survival by a joint classification of the APOE c4 genotype and meat intake (Graph **A**), fish intake (Graph **B**), and egg intake (Graph **C**).

Figure 1 shows the Kaplan-Meier curve of the estimated proportion of survival jointly classified by *APOE* ε4 genotype and meat, fish and egg intake in all participants separately. *APOE* ε4 carriers with high meat, fish or egg intake all had a greater probability of survival compared with APOE ε4 carriers with low intake of the respective food types (*p* < 0.001 for all). However, for APOE ε4 non-carriers, little difference in survival was found among different intake levels of meat, fish and egg.

**Figure 2:**
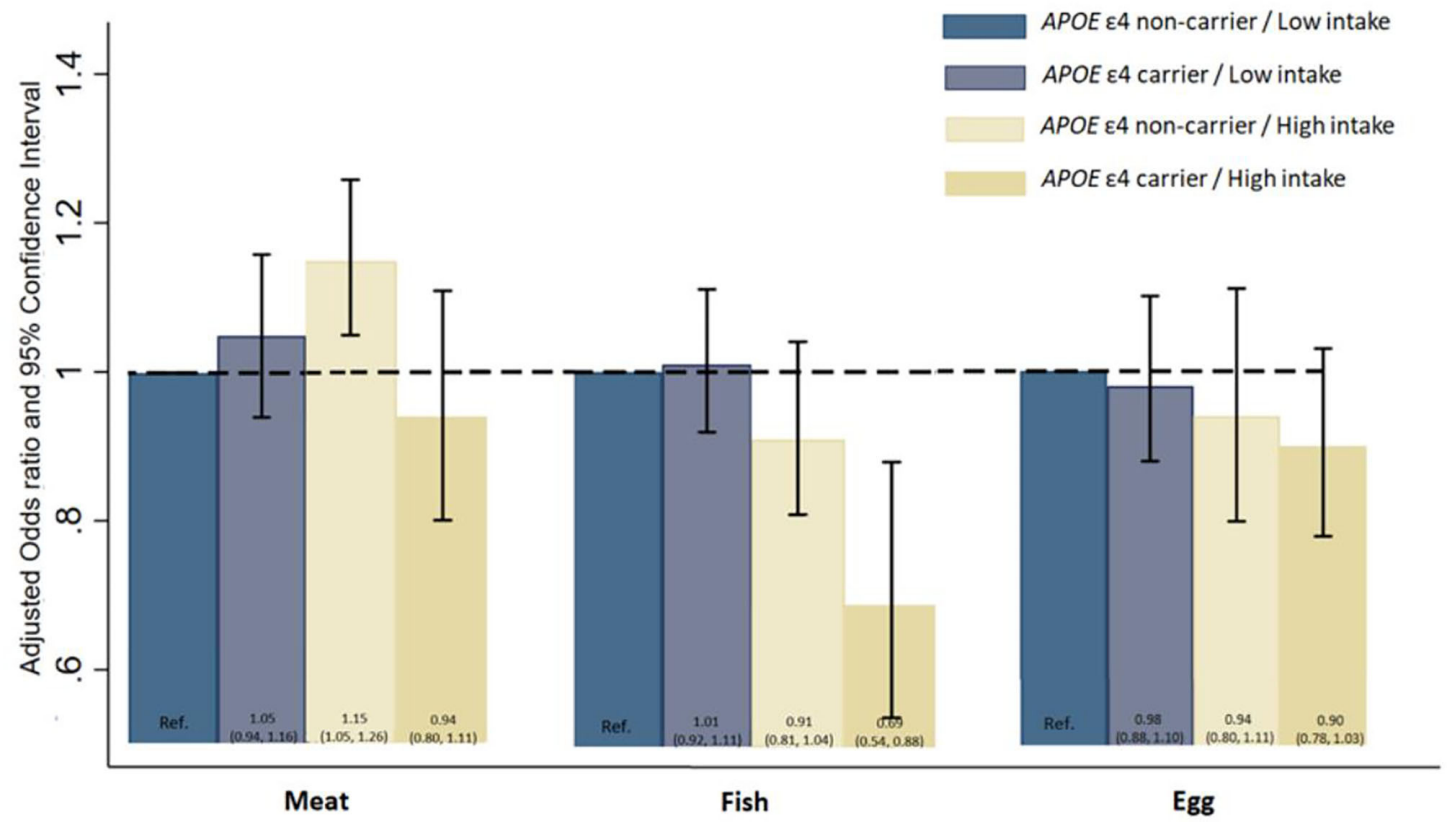
Hazard ratios and 95% Confidence Interval for all-cause mortality by a joint classification of the *APOE* E4 genotype and meat, fish, and egg intake. Model was adjusted for age, sex, education, marital status, ethnicity, living arrangement, residence, occupation, other nine kind of dietary components tobacco smoking status, alcohol drinking status, current physical activity, abnormal body weight, activity of daily living, leisure activity score, mini-mental state examination score, and eight kinds of self-reported diseases (COPD, tuberculosis, cancer, cataract, glaucoma, diabetes, stroke, and cardiovascular disease).

Figure 2 presents the hazard ratios for all-cause mortality from three separate models by a joint classification of the *APOE* ε4 genotype and meat, fish and egg consumption, adjusting for the full set of covariates as in Model 3. Compared with *APOE* ε4 non-carriers with low intake of meat, the all-cause mortality of *APOE* ε4 non-carriers with high intake of meat is significantly higher (HR: 1.15, 95% CI: 1.05, 1.26). Compared with *APOE* non-carriers with low intake of fish, *APOE* ε4 carriers with high intake of fish was found to have substantially lower hazard of death (HR: 0.69, 95% CI: 0.54, 0.88).

**Figure 3:**
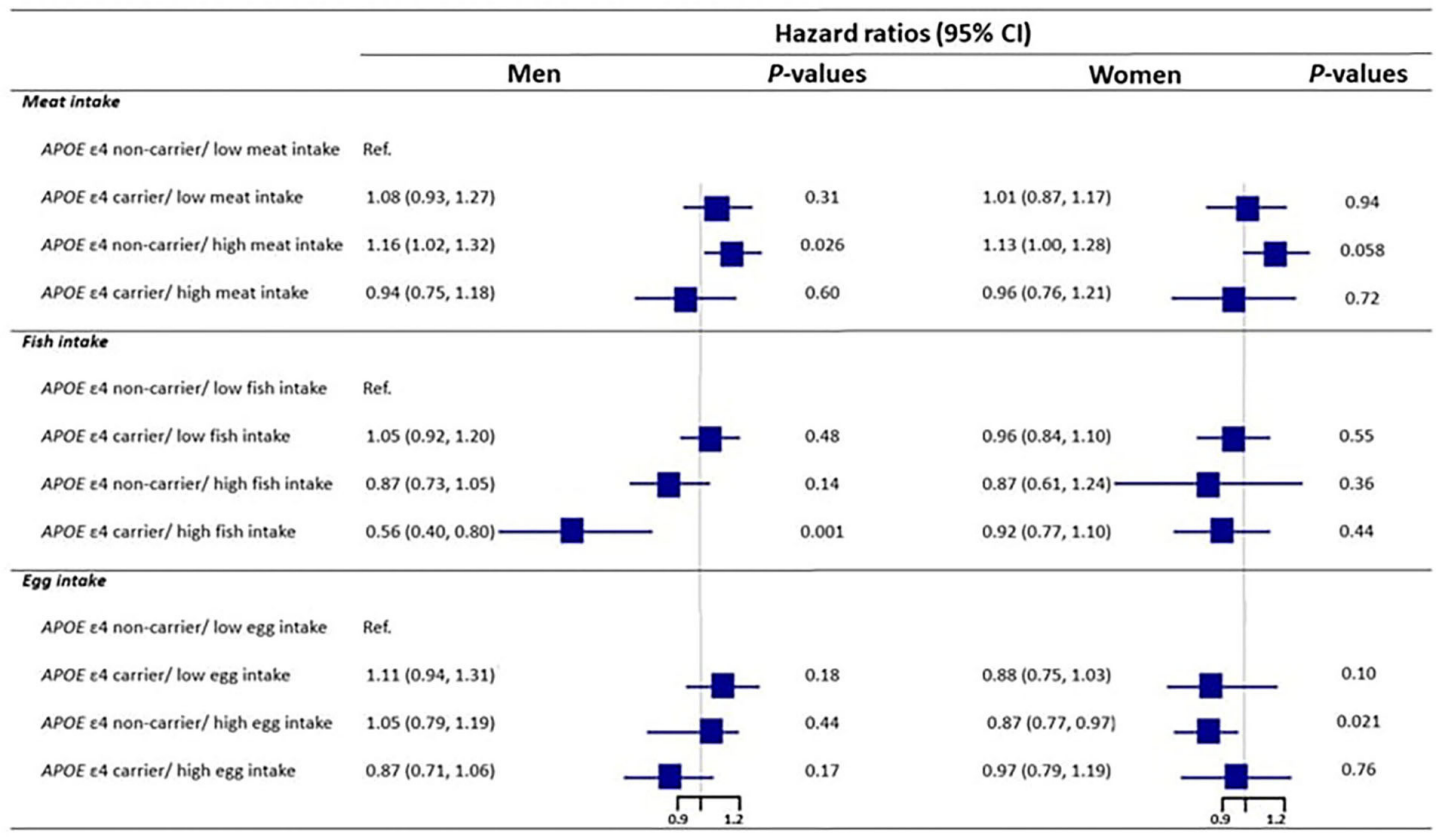
The interaction of *APOE* E4 genotype with meat, fish, and egg on mortality by sex. Model was adjusted for age, sex, education, marital status, ethnicity, living arrangement, residence, occupation, other nine kind of dietary components tobacco smoking status, alcohol drinking status, current physical activity, abnormal body weight, activity of daily living, leisure activity score, mini-mental state examination score, and eight kinds of self-reported diseases (COPD, tuberculosis, cancer, cataract, glaucoma, diabetes, stroke and cardiovascular disease).

We further conducted subgroup analyses by sex to investigate the associations between the joint classifications of *APOE* genotype and food intake and all-cause mortality (Figure 3). We conducted the subgroup analyses with the three food types separately, with the reference group being “*APOE* ε4 non-carrier with low intake of each food type.” Among male, significant interactions between *APOE* genotype and meat, fish, and egg intake on mortality were observed (*P* = 0.037, 0.013, and 0.023 for meat, fish, and egg, separately). However, these interactions were not significant among the female. For meat intake, among male, *APOE* non-carriers with high meat intake had significantly higher mortality when compared with the associated reference group (HR: 1.16, 95% CI: 1.02, 1.32), which is consistent with the analysis among all participants. Among female, however, the difference was only marginally significant (HR: 1.13, 95% CI: 1:00, 1.28). For fish intake, in male, the all-cause mortality was substantially lower among *APOE* ε4 carrier with high fish intake compared with the reference group (HR = 0.56, 95% CI: 0.40, 0.80), but no similar result was found in the female. For egg intake, among male, none of the joint classifications had significantly results, but among female, *APOE* ε4 non-carrier with high egg intake had significantly lower mortality than the reference group (HR = 0.87 CI: 0.77, 0.97).

Supplementary Table 2 showed the stratified analysis by *APOE* ε4 genotype and meat, fish and egg consumption. Significant interaction between *APOE* ε4 genotype and meat and fish consumption were observed (*P* < 0.05 for both) but not for egg consumption (*P* = 0.70). Among *APOE* ε4 non-carriers, meat consumption was linked to an increased risk of death, but the association between fish intake and mortality was not significant (high vs. Low: meat intake: HR: 1.13, 95% CI: 1.04, 1.25; fish intake: HR: 0.90, 95% CI: 0.79, 1.02). Among *APOE* ε4 carriers, high fish intake was associated with lower mortality, but the association between meat intake and mortality was attenuated (high vs. Low: meat intake: HR: 0.91, 95% CI: 0.75, 1.12; fish intake: HR: 0.74, 95% CI: 0.56, 0.98).

### Sensitivity Analyses

In the sensitivity analyses, we present the associations between meat, fish, and egg intake, and all-cause mortality after excluding deaths during the first year (Supplementary Table 3), excluding participants with severe comorbidities (Supplementary Table 4), and additionally adjusting for BMI (Supplementary Table 5). The results are consistent with the main finding: for all three scenarios, in model 3, higher meat intake was significantly associated with higher risk of mortality, lower fish intake was associated with higher risk of mortality, while egg intake had no significant association with mortality risk (Supplementary Tables 3–5).

## Discussion

In this prospective cohort study of older adults aged 65 or older, we found that higher meat intake and lower fish intake increased the risk of mortality. We found significant interaction between *APOE* genotype and meat and fish intake. *APOE* ε4 non-carriers with high meat intake had a higher risk of mortality and *APOE* ε4 carriers with high fish intake had a lower risk of mortality compared with *APOE* ε4 non-carriers with low meat or fish intake, separately. Interestingly, such relationship was restricted to the male and was not observed in the female.

The associations between each dietary component and mortality found in our study were consistent with previous meta-analysis (Meat: positively associated, Fish: negatively associated, Egg: not associated) (20–23). For the association between egg intake and health, which had some controversies in the existing literatures (21, 22), our results reported in Table 2 added to the evidence that no significant association could be found between egg consumption and mortality among the Chinese elderly.

When examining the relationship between meat, fish and egg intake and mortality, stratified by *APOE* ε4 genotype, we observed that the inverse association between fish intake and mortality was only significant among *APOE* ε4 carriers, and the increased risk of high meat intake was only observed among non-carriers. We are not aware of any other studies that touched on this relationship in terms of mortality. However, In several observational studies, the *APOE* ε4 genotype and other modifiable factors have a similar interactive relationship in their impact on cognitive function. According to one study, *APOE* ε4 carriers had a lower rate of cognitive function decline with higher weekly seafood consumption, while among the *APOE* ε4 non-carriers, the association of cognitive function decline and seafood consumption was non-significant (24). Furthermore, some studies on the interaction between *APOE* ε4 genotype and modifiable risk factors (25–27) on dementia found significant results, with the effects of these favorable factors being more pronounced among *APOE* 4 carriers than the non-carriers, which echoes our findings. The possible explanation is that carrying *APOE* ε4 may amplify the benefits of modifiable factors, such as high intake of fish and low intake of meat. It is probable that carrying *APOE* ε4 increases the benefits of favorable modifiable factors such as a high fish and a low meat intake of our study. But, the mechanism of this interaction is not fully understood, and further investigations is needed.

Another plausible explanation for the observed gene-by-diet interaction may be the interaction between *APOE* genotype and dietary pattern on lipid-related biomarkers and cholesterol (10–12). *APOE* is a multifunctional protein which transports and delivers cholesterol and other lipids in the plasma via binding to cell surface apoE receptors (28). Carriers of the ε4 allele of the *APOE* gene have higher total and low-density lipoprotein cholesterol levels than non-carriers (29–33).

In another trial, it was observed that after replacing dietary saturated fats with low glycemic index carbohydrates for 6 months, *APOE* ε4 carriers showed a higher decrease rate in total cholesterol and apo B than non-carriers, which included 389 participants with 125 *APOE* ε4 carriers (12). In the SATgenε study including 22 *APOE* ε4 carriers out of 47 participants, it was suggested that *APOE* ε4 carriers had a greater variation of several biomarkers such as fasting triglycerides and C-reactive protein to dietary fat manipulation in comparison with *APOE* ε4 non-carriers, although no effect of this allelic profile on cholesterol concentrations was observed (12). Additionally, the *APOE* genotype can also modify the effect of dietary fat manipulation on the plasma long-chain polyunsaturated fatty acids (10). Generally, it was suggested that *APOE* ε4 carriers had a higher sensitivity of those lipid-related biomarkers to dietary pattern change in the short-term interventional studies. Thus, it is possible that the *APOE* genotype may modify the effect of meat and fish intake accompanied with rich lipid on long-term outcomes by varying response of lipid-related biomarkers. Additionally, the interaction between *APOE* ε4 genotype and fish intake may be also attributable to the higher dietary n-3 fatty acids, plasma eicosapentaenoic acid (EPA) and docosahexaenoic acid (DHA) with higher fish intake, and *APOE* ε4 genotype may interact with those biomarkers. Previous study showed that the associations of higher dietary n-3 fatty acids (24), EPA and DHA (34) with slower cognitive decline are more pronounced among *APOE* ε4 carriers compared with ε4 non-carriers. However, there is still a lack of evidence on the modifying effect of *APOE* genotype on those biomarkers in response to a changed dietary pattern among the Chinese older population with a significantly lower average EPA, DHA, lipid and cholesterol levels (35) compared with the Western population, and further interventional studies were warranted.

We also found that this gene-by-diet interaction was restricted to the male, and this might be explained by the impacts of *APOE* genotype on some biomarkers. An observational study included 4,410 American found that the relationship between *APOE* genotype and plasma lipid levels was only significant among females (36) and similarly, the relationship between carbohydrate intake and plasma HDL-C levels was only significant among females *APOE* ε4 carriers (31). Additionally, in an interventional study of fish oil supplement, the triacylglycerol-lowering responses was found greater in male with *APOE* ε4 allele (37). Some other studies also indicated that the impact of *APOE* genotype on health outcomes also varied by sex. For instance, the *APOE* ε4 alleles had a larger negative influence on the onset of AD in females than in males, and the link between *APOE* genotype and various AD-related biomarkers was only significant in females (38). Although it is still unclear for which sex the *APOE* ε4 allele was more sensitive in response to different dietary pattern, the sex-specific interaction can be partially explained by those prior evidence.

Our findings are important for two reasons. First, the identification of this diet-gene interactions offers an opportunity to study dietary interventions from a genetic perspective. If our findings could be further validated by large-scale interventional studies, meaningful implications could be generated to develop personalized dietary interventions that consider, or maybe even take advantage of, the effects of genetic factors. Second, APOE genotyping was currently widely used in clinical studies and is included in many commercial genetic testing products. Exploration of this specific genotype is thus of high public interests and may contribute to wider public health impact.

Our study has several strengths. The sample size was large (*n* = 8,506 with 1,640 *APOE* ε4 carriers) with long follow-up duration (longest over 14 years). We performed multivariable Cox regression models adjusted by demographics characteristics and many potential confounders and several sensitivity analyses to evaluate the robustness of the results. Our study also has some limitations. First, the measure of dietary components was self-reported, without detailed quantitative dietary intake evaluation and is subject to recall bias. We provided several existing references that validated our measurement (15–17). Second, some potentially relevant dietary information was not collected, including the nuts, oil, and total caloric intake, which made it impossible to adjust for total energy intake in the analyses. We tried to compensate for this gap by considering many key determinants of energy intake such as age, sex, BMI, comorbidities and physical activity, and we encourage future studies to further investigate these factors. Last but not least, due to the nature of cohort study, we are cautious in providing clinical recommendations, but we call for large-scale rigorous trials to provide more definitive evidence to the interactive effects between *APOE* ε4 allele and dietary factors on mortality in the elderly population.

## Conclusion

In summary, we found that *APOE* genotype can modify the associations of meat and fish intake with mortality. Further research is needed to replicate our results in other populations and other dietary factors. To further confirm the potential causality among our studies variables, future studies on molecular pathways and large-scale interventional studies are warranted. If substantiated with further evidence, our findings may contribute to personalized dietary recommendation and bring more health benefits for the elderly in this era of precision health.

## Data Availability Statement

The original contributions presented in the study are included in the article/Supplementary Material, further inquiries can be directed to the corresponding author/s.

## Ethics Statement

The studies involving human participants were reviewed and approved by The Research Ethics Committees of Peking University and Duke University. The patients/participants provided their written informed consent to participate in this study.

## Author Contributions

LY and XJ: concept and design. XJ and CY: acquisition, analysis, or interpretation of data. XJ, LY, and YL: drafting of the manuscript. LY, SX, EG, XZ, and ZN: critical revision of the manuscript for important intellectual content. XJ and YY: statistical analysis. YZ and LY: obtained funding. LY: administrative, technical, or material support. All authors contributed to the article and approved the submitted version.

## Conflict of Interest

XJ, XZ, and ZN are the employees of MindRank AI. The remaining authors declare that the research was conducted in the absence of any commercial or financial relationships that could be construed as a potential conflict of interest.
