# Supplementary material for: Apolipoprotein E Genotype, Meat, Fish, and Egg Intake in Relation to Mortality Among Older Adults: A Longitudinal Analysis in China
Source: Front Med (Lausanne). 2021 Jul 20;8:697389. doi: 10.3389/fmed.2021.697389 (PMC8329349; doi:10.3389/fmed.2021.697389)
Supplement: Supplementary file 1 [file Data_Sheet_1.docx]

**Appendix:**

**Table S1.**

**Stratified analysis: The interaction between APOE ε4 genotype with meat, fish and Egg on mortality**

| **Food consumption** | **APOE genotype** | **Multivariable adjusted Cox model ^a^** | | ***P* for interaction ^b^** |
| --- | --- | --- | --- | --- |
|  |  | **Hazard ratios (95% CI)** | **P-values** |  |
| ***Meat intake*** | ***APOE* ε4 non-carrier** |  |  | 0.015 |
|  | Low | *Ref.* |  |  |
|  | High | 1.13 (1.04, 1.25) | 0.007 |  |
|  | ***APOE* ε4 carrier** |  |  |  |
|  | Low | *Ref.* |  |  |
|  | High | 0.91 (0.75, 1.12) | 0.38 |  |
| ***Fish intake*** | ***APOE* ε4 non-carrier** |  |  | 0.036 |
|  | Low | *Ref.* |  |  |
|  | High | 0.90 (0.79, 1.02) | 0.11 |  |
|  | ***APOE* ε4 carrier** |  |  |  |
|  | Low | *Ref.* |  |  |
|  | High | 0.74 (0.56, 0.98) | 0.046 |  |
| ***Egg intake*** | ***APOE* ε4 non-carrier** |  |  | 0.70 |
|  | Low | *Ref.* |  |  |
|  | High | 0.94 (0.86, 1.03) | 0.17 |  |
|  | ***APOE* ε4 carrier** |  |  |  |
|  | Low | *Ref.* |  |  |
|  | High | 0.89 (0.74, 1.08) | 0.25 |  |

^a^ Model was adjusted for age, sex, education, marital status, ethnicity, living arrangement, residence, occupation, other nine kind of dietary components tobacco smoking status, alcohol drinking status, current physical activity, abnormal body weight, activity of daily living, leisure activity score, mini-mental state examination score, and eight kinds of self-reported diseases (COPD, tuberculosis, cancer, cataract, glaucoma, diabetes, stroke and cardiovascular disease )

^b^ The *P*s for multiplicative interactions between *APOE* ɛ4 genotype and meat, fish and egg intake on their effects in all-cause mortality were tested by adding cross-product terms in the non-stratified Cox regression models.

**Table S2.**

**Characteristics of the 8,506 Participants by *APOE* Genotype and Sex**

| **Characteristics ^a^** | **Total sample**  **N=8,506** | **Male** | |  | | **Female** | | ***P-values*** |
| --- | --- | --- | --- | --- | --- | --- | --- | --- |
|  |  | **ɛ4 non-carrier**  **N=3,240** | **ɛ4 carrier**  **N=815** | | ***P-values*** | **ɛ4 non-carrier**  **N=3,206** | **ɛ4 carrier**  **N=825** |  |
| **Age**, mean ± SD | 81.7 ± 11.7 | 79.3 (10.5) | 79.2 (10.4) | | 0.76 | 84.2 (12.3) | 82.3 (11.8) | <0.001 |
| **Residence** |  |  |  | | 0.55 |  |  | 0.008 |
| Urban | 2,906 (34.2) | 1153 (35.6) | 281 (34.5) | |  | 1167 (32.2) | 305 (37.0) |  |
| Rural | 5,600 (65.8) | 2087 (64.4) | 534 (65.5) | |  | 2459 (67.8) | 520 (63.0) |  |
| **Main occupation before age 60** |  |  |  | | 0.97 |  |  | 0.32 |
| Non-manual | 652 (7.7) | 435 (13.4) | 109 (13.4) | |  | 84 (2.3) | 24 (2.9) |  |
| Manual | 7,854 (92.3) | 2805 (86.6) | 706 (86.6) | |  | 3542 (97.7) | 801 (97.1) |  |
| **Education years** |  |  |  | | 0.22 |  |  | 0.26 |
| None (0) | 4,910 (57.7) | 1056 (32.6) | 284 (34.8) | |  | 2920 (80.5) | 650 (78.8) |  |
| Ever (≥1) | 3,596 (42.3) | 2184 (67.4) | 531 (65.2) | |  | 706 (19.5) | 175 (21.2) |  |
| **Marital status** |  |  |  | | 0.91 |  |  | 0.077 |
| Married (spouse alive) | 3,685 (43.3) | 1949 (60.2) | 488 (60.0) | |  | 996 (27.5) | 252 (30.5) |  |
| Others ^c^ | 4,817 (56.6) | 1290 (39.8) | 326 (40.0) | |  | 2628 (72.5) | 573 (69.5) |  |
| **Ethnicity** |  |  |  | | 0.45 |  |  | 0.95 |
| Han | 7,975 (93.8) | 3051 (94.2) | 773 (94.8) | |  | 3382 (93.3) | 769 (93.2) |  |
| Others (minority) | 531 (6.2) | 189 (5.8) | 42 (5.2) | |  | 244 (6.7) | 56 (6.8) |  |
| **Living arrangement** |  |  |  | | 0.76 |  |  | 0.043 |
| With household member | 7,118 (83.7) | 2804 (86.5) | 702 (86.1) | |  | 2922 (80.6) | 690 (83.6) |  |
| Alone | 1,388 (16.3) | 436 (13.5) | 113 (13.9) | |  | 704 (19.4) | 135 (16.4) |  |
| **Impaired activity of daily living** ^d^ |  |  |  | | 0.52 |  |  | 0.38 |
| Yes | 1,109 (13.0) | 271 (8.4) | 74 (9.1) | |  | 631 (17.4) | 133 (16.1) |  |
| No | 7,395 (86.9) | 2967 (91.6) | 741 (90.9) | |  | 2995 (82.6) | 692 (83.9) |  |
| **Leisure activities** ^e^, mean ± SD | 12.2 ± 2.9 | 12.7 (2.8) | 12.8 (2.9) | | 0.75 | 11.6 (2.9) | 12.0 (2.8) | 0.003 |
| **MMSE score**, mean ± SD | 24.0 ± 8.1 | 26.0 (6.5) | 25.7 (7.0) | | 0.21 | 22.1 (9.0) | 22.8 (8.4) | 0.064 |
| **Tobacco smoking status** |  |  |  | | 0.24 |  |  | 0.64 |
| Ever | 2,879 (33.8) | 1898 (58.6) | 496 (60.9) | |  | 391 (10.8) | 94 (11.4) |  |
| Never | 5,627 (66.2) | 1342 (41.4) | 319 (39.1) | |  | 3235 (89.2) | 731 (88.6) |  |
| **Alcohol drinking status** |  |  |  | | 0.56 |  |  | 0.41 |
| Ever | 2,518 (29.6) | 1569 (48.4) | 404 (49.6) | |  | 451 (12.4) | 94 (11.4) |  |
| Never | 5,988 (70.4) | 1671 (51.6) | 411 (50.4) | |  | 3175 (87.6) | 731 (88.6) |  |
| **Abnormal body weight** |  |  |  | | 0.016 |  |  | 0.84 |
| No | 7,844 (92.2) | 2939 (90.7) | 761 (93.4) | |  | 3375 (93.1) | 769 (93.2) |  |
| Yes | 662 (7.8) | 301 (9.3) | 54 (6.6) | |  | 251 (6.9) | 56 (6.8) |  |
| **Regular physical activity** |  |  |  | | 0.26 |  |  | 0.29 |
| No | 5,901 (69.4) | 2042 (63.0) | 531 (65.2) | |  | 2723 (75.1) | 605 (73.3) |  |
| Yes | 2,605 (30.6) | 1198 (37.0) | 284 (34.8) | |  | 903 (24.9) | 220 (26.7) |  |
| **Meat intake** |  |  |  | | 0.91 |  |  | 0.25 |
| Rarely or never | 1,084 (12.7) | 351 (10.8) | 92 (11.3) | |  | 512 (14.1) | 129 (15.6) |  |
| Occasionally | 4,616 (54.3) | 1747 (53.9) | 434 (53.3) | |  | 1976 (54.5) | 459 (55.6) |  |
| Everyday | 2,806 (33.0) | 1142 (35.2) | 289 (35.5) | |  | 1138 (31.4) | 237 (28.7) |  |
| **Fish intake** |  |  |  | | 0.88 |  |  | 0.64 |
| Rarely or never | 1,924 (22.6) | 638 (19.7) | 166 (20.4) | |  | 906 (25.0) | 214 (25.9) |  |
| Occasionally | 5,299 (62.3) | 2077 (64.1) | 515 (63.2) | |  | 2217 (61.1) | 490 (59.4) |  |
| Everyday | 1,283 (15.1) | 525 (16.2) | 134 (16.4) | |  | 503 (13.9) | 121 (14.7) |  |
| **Egg intake** |  |  |  | | 0.35 |  |  | 0.16 |
| Rarely or never | 1,021 (12.0) | 349 (10.8) | 102 (12.5) | |  | 468 (12.9) | 102 (12.4) |  |
| Occasionally | 4,120 (48.4) | 1558 (48.1) | 379 (46.5) | |  | 1754 (48.4) | 429 (52.0) |  |
| Everyday | 3,365 (39.6) | 1333 (41.1) | 334 (41.0) | |  | 1404 (38.7) | 294 (35.6) |  |

Abbreviation: MMSE: Mini-Mental State Examination

^a^ Numbers shown are N (%) unless otherwise noted.

^c^ Other marital status includes separated, widowed, divorced, never married.

^d^ Activity of daily living: assessed by six self-reported questions: “Do you need assistance in bathing/dressing/toileting/transferring/eating/continence?”. Impaired ADL was defined as if the participants answered ‘Yes’ for any of those questions.

^e^ Leisure activity score: eight activities are assessed: visiting neighbors, shopping, cooking, washing clothes, walking 1 km, lifting 5 kg, crouching and standing up three times, and taking public transportation. We scored each activity 1 for ‘never’, 2 for ‘sometimes’ 3 for ‘almost every day’. The score ranged from 5 to 21 with a higher score indicating more leisure activities.

**Table S3.**

**Sensitivity Analysis: Hazard Ratios and 95% Confidence Intervals (CIs) of Meat, Fish, Egg and Animal Protein Intake after Excluding the Death in the First Year (N=355)**

| **Groups** | **Participants**  **(N=8,151)** | **Hazard Ratio (95% Confidence Interval)** | | | |  |  |  |
| --- | --- | --- | --- | --- | --- | --- | --- | --- |
|  |  | **Age-sex adjusted model** | **Model 1*** | **Model 2**** | **Model 3***** |  |  |  |
| **Meat intake** |  |  |  |  |  |  |  |  |
| Rarely or never | 1,846 | Ref. |  |  |  |  |  |  |
| Occasionally | 5,058 | 0.91 (0.82, 1.00) | 1.05 (0.96, 1.12) | 1.06 (0.94, 1.21) | 1.09 (1.00, 1.23) |  |  |  |
| Everyday | 1,247 | 0.90 (0.77, 1.02) | 1.05 (0.94, 1.13) | 1.05 (0.92, 1.16) | 1.09 (0.96, 1.20) |  |  |  |
| **Fish intake** |  |  |  |  |  |  |  |  |
| Rarely or never | 1,056 | Ref. | | | |  |  |  |
| Occasionally | 4,397 | 0.74 (0.51, 0.95) | 0.83 (0.59, 0.83) | 0.83 (0.59, 1.04) | 0.84 (0.60,1.05) |  |  |  |
| Everyday | 2,698 | 0.63 (0.43, 0.80) | 0.72 (0.51, 0.72) | 0.73 (0.52, 0.89) | 0.77 (0.56,0.94) |  |  |  |
| **Egg intake** |  |  |  |  |  |  |  |  |
| Rarely or never | 1,056 | Ref. | | | |  |  |  |
| Occasionally | 4,397 | 1.15 (1.03, 1.30) | 1.08 (0.99, 1.18) | 1.06 (0.93, 1.14) | 1.07 (0.98, 1.19) |  |  |  |
| Everyday | 2,698 | 1.13 (1.04, 1.28) | 0.98 (0.87, 1.10) | 1.04 (0.85, 1.11) | 1.01 (0.88, 1.21) |  |  |  |

Model 1* adjusted for demographic variables: age, sex, education, marital status, ethnicity, living arrangement, residence, and occupation and other nine kind of dietary components; Model 2** added lifestyle variables (tobacco smoking status, alcohol drinking status and current physical activity) and abnormal body weight for adjustment; Model 3*** further adjusted for activity of daily living, leisure activity score, The Mini-Mental State Examination (MMSE) score, and eight kinds of self-reported diseases (COPD, tuberculosis, cancer, cataract, glaucoma, diabetes, stroke and cardiovascular disease ) on the basis of Model 2. For the models including dichotomized protein intake index, egg, fish and meat which we built the index from were excluded from the adjustment of dietary components.

**Table S4.**

**Sensitivity Analysis: Hazard Ratios and 95% Confidence Intervals (CIs) of Meat, Fish, Egg and Animal Protein Intake after Excluding the Participants with Comorbidity (N=1,858)**

| **Groups** | **Participants**  **(N=6,648)** | **Hazard Ratio (95% Confidence Interval)** | | | |
| --- | --- | --- | --- | --- | --- |
|  |  | **Age-sex adjusted model** | **Model 1*** | **Model 2**** | **Model 3***** |
| **Meat intake** |  |  |  |  |  |
| Rarely or never | 801 | Ref. |  |  |  |
| Occasionally | 3,615 | 0.98 (0.85, 1.10) | 1.03 (0.98, 1.07) | 1.03 (1.00, 1.07) | 1.04 (0.93, 1.16) |
| Everyday | 2,232 | 1.03 (1.00, 1.06) | 1.12 (0.99, 1.24) | 1.12 (0.99,1.24) | 1.13 (1.01, 1.28) |
| **Fish intake** |  |  |  |  |  |
| Rarely or never | 1,477 | Ref. |  |  |  |
| Occasionally | 4,173 | 0.74 (0.51, 0.95) | 0.83 (0.59, 0.83) | 0.83 (0.59, 1.04) | 0.84 (0.60,1.05) |
| Everyday | 998 | 0.63 (0.43, 0.80) | 0.72 (0.51, 0.72) | 0.73 (0.52, 0.89) | 0.77 (0.56,0.94) |
| **Egg intake** |  |  |  |  |  |
| Rarely or never | 1,011 | Ref. |  |  |  |
| Occasionally | 4,197 | 0.78 (0.59,0.96) | 0.84 (0.61,1.04) | 0.91 (0.74,1.07) | 0.88 (0.69,1.06) |
| Everyday | 2,497 | 0.88 (0.73,1.02) | 0.72 (0.53,0.89) | 1.02 (0.87,1.14) | 0.99 (0.83,1.12) |

Model 1* adjusted for demographic variables: age, sex, education, marital status, ethnicity, living arrangement, residence, and occupation and other nine kind of dietary components; Model 2** added lifestyle variables (tobacco smoking status, alcohol drinking status and current physical activity) and abnormal body weight for adjustment; Model 3*** further adjusted for activity of daily living, leisure activity score, The Mini-Mental State Examination (MMSE) score, and eight kinds of self-reported diseases (COPD, tuberculosis, cancer, cataract, glaucoma, diabetes, stroke and cardiovascular disease ) on the basis of Model 2. For the models including dichotomized protein intake index, egg, fish and meat which we built the index from were excluded from the adjustment of dietary components.

**Table S5.**

**Sensitivity Analysis: Hazard Ratios and 95% Confidence Intervals (CIs) of Meat, Fish, Egg and Animal Protein Intake with additionally adjusting for BMI**

| **Groups** | **Participants**  **(N)** | **Hazard Ratio (95% Confidence Interval)** | |
| --- | --- | --- | --- |
|  |  | **Model 2**** | **Model 3***** |
| **Meat intake** |  |  |  |
| Rarely or never | 801 | Ref. |  |
| Occasionally | 3,615 | 1.10 (0.95, 1.22) | 1.18 (1.03, 1.31) |
| Everyday | 2,232 | 1.08 (0.89, 1.25) | 1.15 (0.96, 1.33) |
| **Fish intake** |  |  |  |
| Rarely or never | 1,477 | Ref. |  |
| Occasionally | 4,173 | 0.58 (0.33, 0.78) | 0.65 (0.41, 0.85) |
| Everyday | 998 | 0.71 (0.43, 0.96) | 0.72 (0.45, 0.96) |
| **Egg intake** |  |  |  |
| Rarely or never | 765 | Ref. | |
| Occasionally | 3,300 | 1.08 (0.90, 1.22) | 1.04 (0.86, 1.19) |
| Everyday | 2,583 | 0.95 (0.73, 1.13) | 0.91 (0.70, 1.11) |

Model 1* adjusted for demographic variables: age, sex, education, marital status, ethnicity, living arrangement, residence, and occupation, other nine kind of dietary components, lifestyle variables (tobacco smoking status, alcohol drinking status and current physical activity) and BMI; Model 2*** further adjusted for activity of daily living, leisure activity score, The Mini-Mental State Examination (MMSE) score, and eight kinds of self-reported diseases (COPD, tuberculosis, cancer, cataract, glaucoma, diabetes, stroke and cardiovascular disease ). For the models including dichotomized protein intake index, egg, fish and meat which we built the index from were excluded from the adjustment of dietary components.
