# Supplementary material for: Apolipoprotein E Genotype, Meat, Fish, and Egg Intake in Relation to Mortality Among Older Adults: A Longitudinal Analysis in China
Source: Front Med (Lausanne). 2021 Jul 20;8:697389. doi: 10.3389/fmed.2021.697389 (PMC8329349; doi:10.3389/fmed.2021.697389)
Supplement: Supplementary file 2 [file Data_Sheet_2.docx]

**Development of the Statistical Analysis Plan**

Study Context

Data for the present study come from the CLHLS study, a longitudinal study since 1998 with follow-up surveys every 2 to 3 years. The CLHLS randomly selected 806 cities and counties in 23 provinces of China using multi-stage stratified sampling. The study areas covered 85% Chinese population. More details on sampling design and data quality can be found elsewhere. The design of CLHLS was approved by the Campus Institutional Review Board of Duke University (Pro00062871) and the Biomedical Ethics Committee of Peking University (IRB00001052-13074). All participants or their legal representatives signed written consent forms during the surveys.

The association between diet and mortality have been well investigated. However, the effects of meat, fish and egg consumption on mortality are not consistent across studies and considerable interindividual heterogeneity has been noted. For instance, a study with 134,290 Chinese adults suggested that a high intake of meat was associated with higher mortality^1^, while a pooled analysis including 29,721 Asians failed to provide similar evidence^2^. One potential explanation for this inconsistency may be the modifying effect of genetic factors across different individuals.

We think APOE ε4 could be one of the specific explanatory genes because a recent study found that APOE ε4 carriers had a faster response in several lipid-related biomarkers to the same dietary interventions compared with the ε4 non-carrier^3^. We think such existing evidence increased the biological plausibility for the possible interactions between APOE genotype and meat, fish and egg intake on human’s health. Therefore, we hypothesized that the relation between meat, fish and egg consumption and mortality may be modified by APOE ε4 genotype among older adults. We thought a good way to test this hypothesis would be examining the effect of *APOE* ε4 genotype on the interaction between meat, fish and egg intake, and all-cause mortality among elderly aged over 65 years old. CLHLS study could provide suitable data because it covers the oldest elderly and there is a genetic sub-study which provided the information of *APOE* genotype. Thus, we conducted this study.

Analysis Plan prior to commencing the present manuscript

The analyses presented in the current manuscript evolved from the following research question and associated statistical analysis:

1. *What is the association between APOE gene and the effect of meat, fish and egg intake on all-cause mortality among the elderly?*
   1. *We will use the pooled baseline cross-sectional data from CLHLS genetic sub-study.*
   2. *The intake frequency of meat, fish and egg will be reported in 3 categories (“almost every day”, “sometimes or occasionally”, or “rarely and never”) at baseline.*
   3. *The APOE genotype will be group by whether the participants carrying APOE ε4 allele (ε2/ε4, ε3/ε4, ε4/ε4 genotypes) or not (ε2/ε2, ε2/ε3, ε3/ε3)*
2. *What is the association between meat, fish and egg intake and all-cause mortality?*
   1. *We will use cox regression model to calculate the hazard ratios for all-cause mortality.*
3. *What is the interaction of APOE genotype with meat, fish and egg intake and on all-cause mortality?*
   1. *Examine the effect modification of APOE genotype on the association of meat, fish and egg intake and all-cause mortality by subgroup analysis*
   2. *The intake frequency of meat, fish and egg will be reported in 2 categories (“almost every day” will be defined as “high intake”, “Sometimes or occasionally” and “rarely or never” will be merged into “low intake”) in subgroup analysis.*

The following revisions to the analysis plan were made:

1. Sensitivity analyses: excluding deaths in the first year after the follow-up survey, as these early decedents may suffer from serious conditions that might confound the relationship
2. Sensitivity analyses: excluding participants with baseline comorbidity (including heart disease, stroke, respiratory disease, and cancer)
3. Sensitivity analyses: performing the same analysis on data from the 2008 and 2012 waves, but replaced the body weight variable with body mass index (BMI, height was firstly measured at the 2008 wave)
4. Sensitivity analyses: excluding participants who had different dietary patterns of meat, fish and egg intake between the baseline wave and when they were 60 years old (e.g. high intake at 60 years old and low intake at baseline, or the other way around)
5. Sensitivity analyses: excluding participants who had severe cognitive impairment at baseline to reduce the recall bias

**Reference**

1. Takata Y, Shu XO, Gao YT, et al. Red meat and poultry intakes and risk of total and cause-specific mortality: results from cohort studies of Chinese adults in Shanghai. *PLoS One.* 2013;8(2):e56963. <https://doi.org/10.1371/journal.pone.0056963>

2. Lee JE, McLerran DF, Rolland B, et al. Meat intake and cause-specific mortality: a pooled analysis of Asian prospective cohort studies. *Am J Clin Nutr.* 2013;98(4):1032-1041. <https://doi.org/10.3945/ajcn.113.062638>

3. Deelen J, Evans DS, Arking DE, et al. A meta-analysis of genome-wide association studies identifies multiple longevity genes. *Nat Commun.* 2019;10(1):3669. <https://doi.org/10.1038/s41467-019-11558-2>
